# Multiplexed rectangular dielectric gratings with multiple narrow-band refractive index filtering and sensing

**DOI:** 10.1038/s41598-024-52805-x

**Published:** 2024-01-29

**Authors:** Aibibula Abudula, Paiziliya Maitiaximu, Abulizi Abulaiti, Melike Mohamedsedik, Arzigul Rahmut, Feng Xu, Paerhatijiang Tuersun

**Affiliations:** 1 College of Xinjiang Uyghur Medicine, Hoten, 848000 China; 2 Xinjiang Key Laboratory of Hotan Characteristic Chinese Traditional Medicine Research, Hoten, 848000 China; 3 Engineering Research Center of Xinjiang Hotan Traditional Chinese and Ethnic Medicine, Hoten, 848000 China; 4 College of Physics and Information Technology, Shaanxi Normal University, Xi’an, 710062 China; 5 Aksu Vocational and Technical College, Aksu, 843000 China; 6 Department of Physics &, Jiujiang Research Institute, Xiamen University, Xiamen, 361005 China; 7 School of Physics and Electronic Engineering, Xinjiang Normal University, Urumqi, 830054 China

**Keywords:** Optical sensors, Nanophotonics and plasmonics, Metamaterials

## Abstract

We propose a low-loss compound structure consisting of a multiplexed rectangular dielectric grating and a waveguide layer, which can function as multi-band optical filters and sensors in TE and TM polarization by utilizing the resonant mode of the waveguide (WG) and the hybrid SP, respectively. By manipulating the parameters and subsequently constraining the local density of multi-resonant modes to several distinct resonant wavelengths, we propose a novel category of highly sensitive refractive index sensing platforms. Spectral shifts ranging from 110 to 131 nm/RIU with FOM of (22, 26.2)/RIU under TE polarization and 80 to 114 nm/RIU with FOM of (5.7, 8.1)/RIU under TM polarization can be accurately discerned for multiple individual analytes across a broad spectral range. The proposed structures offer enhanced flexibility in the design of structures across a wide spectral range, catering to various potential applications in multi-band optical filters, sensors, and photodetectors.

## Introduction

The gratings have demonstrated exceptional optical properties and have been utilized not only for optical filtering and sensing, but also for manipulating light-matter interactions by modifying the photonic mode densities through their resonance modes^1,2^. Consequently, extensive research has been conducted to showcase their potential as biomedical sensor tools^3–5^. Most of these electromagnetic optical resonances excited by metal optical nanostructures can achieve selective excitation or emission at specific wavelengths within the structure. For instance, they can be used as selective thermal emitters^6–8^, grating-based perfect optical absorption structures^4,9,10^, or tunable plasmonic near-perfect selective absorbers^11,12^. In these all-metal light-matter interaction structures, the excitation of surface plasmon resonance leads to dissipation on the protrusions' surface, resulting in field confinement at the metallic interface and subsequent transfer of a portion of trapped light energy to Ohmic loss. Moreover, it is not feasible for such one-dimensional structures to simultaneously match resonance modes of different orders in the cavity without any Ohmic losses or be excited in both TM and TE polarizations. This is because resonance modes in ordinary cavities/resonators are correlated; their excitation depends on polarization and cannot have independently adjustable resonance positions simply by varying the structure or material parameters^10,13^. Compared to the single resonance modes in typical optical structures, multiplet independently adjustable resonance modes are highly desired for optoelectronic applications with high light-matter interaction, such as multispectral detection^14–16^ and multi-band sensing^5,17–20^. This is due to the inherent limitation of narrow-band properties in single resonance mode structures, which greatly restricts their potential applications. However, single resonance mode structures are not suitable for certain areas such as multispectral detection, sensing, and phase imaging that require distinct optical resonance modes.

Actually, there have been significant research efforts focused on the design of multiband-resonant plasmonic structures for the development of narrow-band filtering or high-sensitivity bio-chemical sensing^21^. Additionally, several reported studies have been dedicated to investigating the near-field coupling of multiple plasmon resonance modes^22^. In general, these structures exhibit intercoupled resonance modes in the near field or are bound by common structural features. Consequently, they are inherently correlated and their resonance positions cannot be independently adjusted^23^.

**Figure 1:**
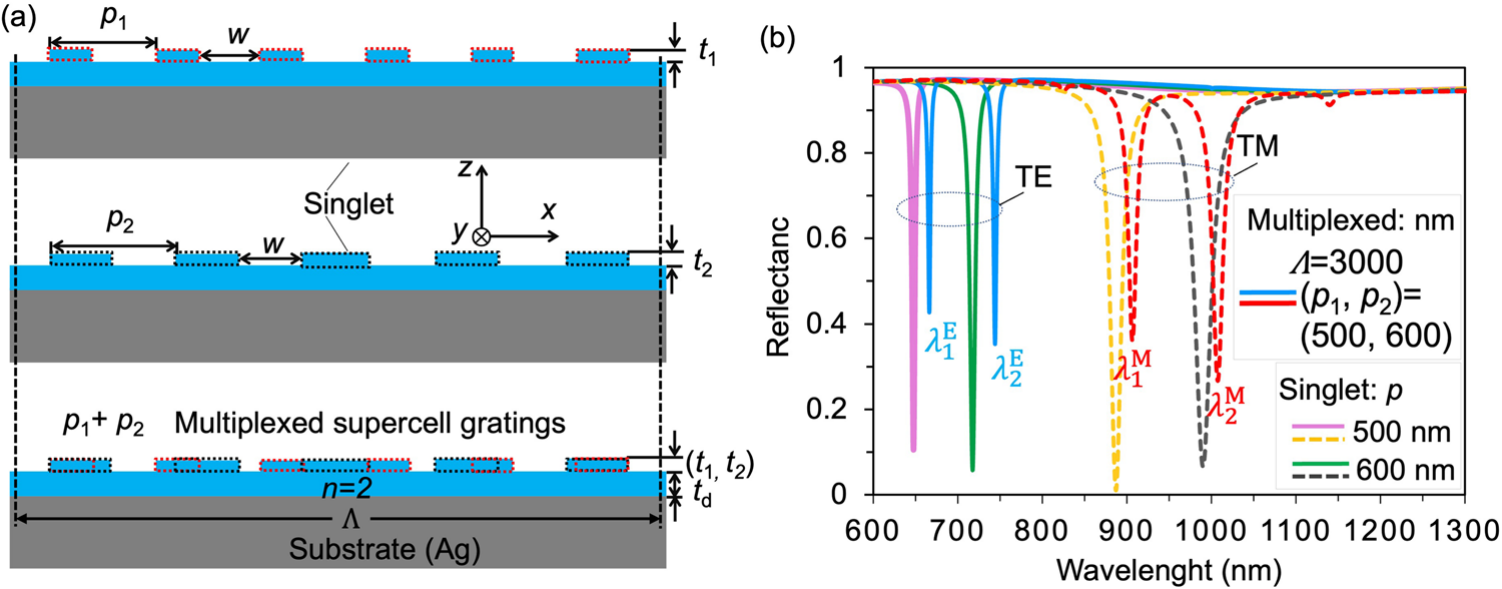
(**a**) Illustration of the surface profile of single-period and multiplexed dielectric rectangular grating with constitutive periods of 500 nm and 600 nm. (**b**) Resonance characteristics in reflection spectra of the grating under normal incidence of TE and TM polarized light, in comparison with those of corresponding singlet gratings.

In this study, we propose a novel multiplexed dielectric grating structure comprising multiple period components on a flat metal substrate sandwiched with a dielectric waveguide layer, as schematically illustrated in Fig. 1a. This design enables the generation of multiple resonance modes with overlapping fields and polarization-selective states to effectively control and enhance light-matter interactions in all directions. The doublet/triplet reflection WG and hybrid SP resonance dips are achieved in the visible-near-infrared region for TE and TM polarization, respectively. The independent multiplet resonance modes exhibit a clear correlation with the parameters of the multiplexed dielectric gratings in both polarizations^24^. Specifically, under TE polarization, both resonance modes experience equal variations due to the presence of WG resonance mode. Although various hybrid SP modes incorporating multi-resonance compound structures have been recently proposed, their independent and arbitrary regulation is hindered by the correlation among resonant modes^25–27^. However, our proposed multi-period composite grating structure exhibits independent and arbitrary tunability under TM polarization due to the presence of mixed SP resonance modes, thereby demonstrating adjustable resonance positions and intensities at each mode. Our focus lies on investigating the properties of multiplet resonance modes such as field distribution, local field enhancement, filtering capabilities, sensing abilities of multiplexed dielectric gratings as well as exploring their dependence on structural dimensions. This allows for flexible design and application catering to various requirements related to controlling and enhancing light-matter interactions. For practical applications, our structure can be combined with existing works such as photoluminescence studies which would provide significant freedom in realizing applicable optical devices capable of simultaneous excitation and emission enhancement. Furthermore, we exploit the strong electromagnetic field enhancement on the structure's surface to examine its infrared sensing performance towards refractive index changes. It is observed that excellent performance can be achieved across all resonances with maximum bulk sensitivity [*nm*/*RIU*] surpassing previous reports by more than 2 times within this range^28^. The proposed multiplexed dielectric grating structures can be applied to potential device in design of multi-band optical filtering, sensing, photoluminescence, and photodetectors.

## Results

### Design and general resonance characteristics of the multiplexed dielectric gratings

The proposed structure is illustrated in Fig. 1a, consisting of rectangular dielectric multiplexed gratings separated by a thin dielectric layer on the Ag substrate. Here we utilize silver as a metal substrate due to its numerous advantages, including high electrical conductivity, exceptional optical properties, low resistance, cost-effectiveness, and minimal imaginary part of relative dielectric constant^29^. Therefore, the selection of silver as the simulation material is primarily based on its outstanding performance characteristics. However, one issue that may arise is the gradual oxidation of silver under specific environmental conditions leading to the formation of a layer of silver oxide (Ag_2_O). Particularly in biochemical applications, the presence of silver oxides can potentially restrict the stability and reliability of optical properties. To address this concern regarding silver oxidation, several solutions can be considered such as exploring alternative materials or applying an antioxidant layer on the surface. This would enable utilization in biochemical applications where high stability and antioxidant properties are crucial. The geometric parameters include a top dielectric grating layer thickness of *t*_1_ = *t*_2_ = 40 nm, an interspaced dielectric layer thickness of *t*_d_ = 100 nm, a width of *w* = 300 nm, and a multiplexed reflective dielectric grating with two constitutive periods *p*_1_ = 500 nm and *p*_2_ = 600 nm. The surface profile is modulated by a supercell package of *Λ* = 2*p*_1_*p*_2_/|*p*_1_-*p*_2_|= 3000 nm. Based on the method described below, we conducted simulations to characterize the resonance properties of the dielectric multiplexed grating's reflection spectrum, as depicted in Fig. 1b. The comparison between this spectrum and those of corresponding single-period gratings is also presented in the same figure. It should be noted that achieving reflection troughs in both TE and TM polarizations presents a challenge for one-dimensional structures. The plasmonic waveguide structure with multiplexed rectangular gratings in Fig. 1b exhibits two closely spaced resonant reflection dips, namely (${\lambda }_{1}^{E}$, ${\lambda }_{2}^{E}$) = (666.1 nm, 743.9) and (${\lambda }_{1}^{M}$, ${\lambda }_{2}^{M}$) = (906.4 nm, 1007.3 nm) nm, for TE and TM polarization respectively. These dips have a full width at half maximum (FWHM) of approximately 5 nm and 14 nm for TE and TM polarization respectively. The quality factor (*QF*) of resonance is approximately 148 for TE polarization and around 71 for TM polarization, which is defined as *QF* = *λ*_res_/*FWHM*. The structures with a single period grating exhibit singlet resonant reflection dip at *p* = 500 and 600 nm, respectively, which closely align with those of the multiplexed structure. Furthermore, Fig. 1b illustrates that the resonant position of the multiplexed compound grating structure exhibits a redshift phenomenon compared to that of the single-period traditional grating due to equivalent refraction caused by different filling factor *f*. This is attributed to the small filling factor *f* for reusing the multiplexed compound grating structure, i.e., *w* < *p* − *w*, as shown in Fig. 1a. Based on effective index expressions (3) and (4) in the text and equation *λ*_*res*_ = *Λn*_*eff*_ related to resonance position and effective index, it can be concluded that multiplexed compound grating structures experience a redshift in their resonance inclination. The results demonstrate that the resonance positions of the doublet can be independently and arbitrarily adjusted by varying the periods of the constitutive rectangular dielectric gratings in the multiplexed structure.

**Figure 2:**
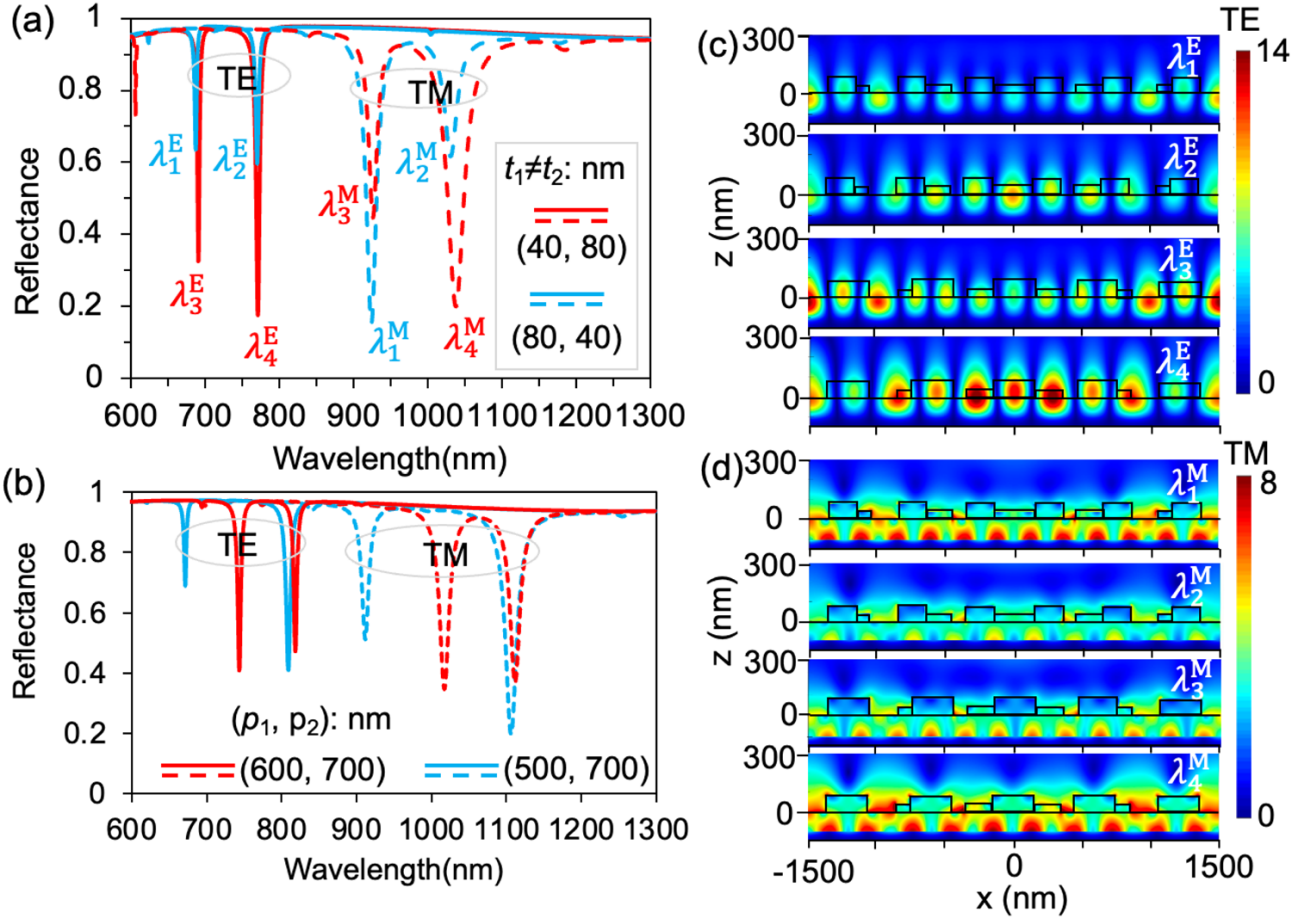
(**a**) Effects of surface fluctuation thickness (e.g., *t*_1_ ≠ *t*_2_) on respective strengths of the doublet resonances for TE and TM polarizations. Here, (*p*_1_, *p*_2_) = (500, 600) nm and *t*_d_ = 100 nm. (**b**) Resonance characteristics in reflection spectra of the multiplexed grating periods of (*p*_1_, *p*_2_) = (600, 700) and (*p*_1_, *p*_2_) = (500, 700) under normal incidence of TE and TM polarized light. (**c-d**) Distributions of the transverse electric field (|*E*|) at the resonance wavelengths of (${\lambda }_{1}^{E}$, ${\lambda }_{2}^{E}$, ${\lambda }_{3}^{E}$, ${\lambda }_{4}^{E}$) and (${\lambda }_{1}^{M}$, ${\lambda }_{2}^{M}$, ${\lambda }_{3}^{M}$, ${\lambda }_{4}^{M}$) for TE and TM polarizations indicated in (**a**), respectively.

Resonance intensities of the multiplet resonance modes determine the degree of confinement in local fields during resonances, which can be quantified by the depths of resonant reflection dips in the spectrum that are subject to modulation strengths imposed by the gratings. Therefore, we investigated how varying fluctuation thickness (*t*_*i*_) of these constitutive gratings affects both resonance modes and their corresponding field distributions. Firstly, we will discuss the impact of surface fluctuation thickness on TE polarization, as illustrated in Fig. 2a. It is observed that, for different values of (*t*_1_, *t*_2_) = (40, 80) and (*t*_1_, *t*_2_) = (80, 40) nm, respectively, depths of the doublet resonant reflection dips at (${\lambda }_{1}^{E}$, ${\lambda }_{2}^{E}$, ${\lambda }_{3}^{E}$, ${\lambda }_{4}^{E}$) = (687.6, 770.4, 691.2, 771.3) nm are observed; there is no effect specific to a single resonant mode; as the thickness of the waveguide layer increases, both resonant modes are affected, resulting in an enhancement of the resonant mode with the smallest shift in its position. Consequently, only the effect of the WG resonant mode is observed here without exciting the SP resonant mode. For the TM polarization, as shown in Fig. 2a, the depths of the doublet resonant reflection dips at (${\lambda }_{1}^{M}$, ${\lambda }_{2}^{M}$, ${\lambda }_{3}^{M}$, ${\lambda }_{4}^{M}$) = (924.5, 1031.3, 926.3, 1038.2) nm can be independently controlled by adjusting the respective thicknesses of the constitutive grating in example cases of (*t*_1_, *t*_2_) = (40, 80) nm and (80, 40) nm ; however, the dip positions remain nearly invariant. Additionally, plasmonic structures with multiplexed gratings of other constitutive periods such as (*p*_1_, *p*_2_) = (500, 700) and (600, 700) nm were simulated as shown in Fig. 2b. The characteristic doublet resonance modes of the example structures for TE and TM polarization are illustrated in Fig. 2b, where corresponding dips in resonance reflection occur at ${\lambda }^{E}$=(671.4, 743.4, 809.1, 818.2) and ${\lambda }^{M}$= (911.8, 1017.1, 1106.5, 1112.1) nm, respectively. The FWHM in TE polarized WG resonance mode exhibits a slightly narrower band compared to that observed in SP hybrid resonance mode. Results show that the local field enhancement at the longer resonance wavelength is larger than that of the shorter one for the longer distance doublet gratings (*p*_1_, *p*_2_) = (500, 700) nm, but slightly smaller for the shorter distance doublet gratings (*p*_1_, *p*_2_) = (500, 700) nm, whether it is TE and TM polarization or not. It is because the higher difference of number of the field nodes 2*Λ*_2_/*p*_2_-2*Λ*_2_/*p*_1_ = 4 at *λ*_1_ (number of field nodes 2*Λ*_2_/*p*_1_ = 14) and *λ*_2_ (number of field nodes 2*Λ*_2_/*p*_2_ = 10) for the former longer distance doublet grating structures of (*p*_1_, *p*_2_) = (500, 700) nm compared with the latter shorter distance doublet grating structures period of (*p*_1_, *p*_2_) = (600, 700) nm with the lower difference of number of the field nodes 2*Λ*_2_/*p*_2_-2*Λ*_2_/*p*_1_ = 2, which results in the light trapped more in the waveguide layer and near the gratings; another possible reason could be the disparity in space ratios between the gratings of different periods^24^. For doublet grating structures with longer distance, characterized by a period of (*p*_1_, *p*_2_) = (500, 700) nm, the space ratio is 25.71%, whereas for shorter distances it is 21.42%. This implies that instead of being diffracted by dielectric gratings, additional light directly transmits to the waveguide layer. The resonance dips exhibit a red shift as the periods increase, owing to the relationship between the resonance wavelength (*λ*_res_) and grating period (*p*), expressed as *λ*_*res*_ = *Λn*_*eff*_^30^. By adjusting the compound grating, it is possible to effectively increase or decrease the required number of resonances and appropriately extend the spectral range, such as into the infrared region. From a resonance perspective, theoretically, the composite structure can support a significant number of resonances. In this embodiment, each period portion of the grating can be finely adjusted to select a specific resonant point. However, excessive resonance for practical applications may lead to unnecessary complexity and potential issues like scattering loss, which could result in reduced or distorted resonance effects. Therefore, for most practical purposes, typically two or three main resonators are selected to strike a balance between efficiency and performance requirements. Regarding tunable resonant wavelengths' dynamic range, changing the grating's period essentially alters its sensitivity towards specific wavelengths. In other words, as the grating period varies so does its corresponding resonant wavelength. This flexibility allows adjusting the dynamic range of resonant wavelengths according to application requirements by properly configuring parameters of the composite grating system—for instance optimizing it for infrared or other specific spectral ranges. Based on simulation results, it can be concluded that multiplexed dielectric rectangular grating structures possess the potential for arbitrary design controllability, enabling independent adjustment with any desired period.

Additionally, we conducted simulations to analyze the field distributions (|*E*|) of the doublet resonance modes in the plasmonic structure, as illustrated in Fig. 2c,d, aiming to demonstrate the impact of variations in thickness of the constitutive grating on local field enhancement. The electric field distribution diagram from Fig. 2c obviously demonstrates that the resonant mode observed under TE polarization can be attributed to the WG resonance mode, with a direct correlation between the thickness of the waveguide layer and the intensity of the electric field. For TE polarization, the field magnitudes at the doublet resonance wavelengths of (${\lambda }_{1}^{{\text{E}}}$, ${\lambda }_{2}^{{\text{E}}}$) and (${\lambda }_{2}^{{\text{E}}}$, ${\lambda }_{4}^{{\text{E}}}$) exhibit nearly equal values due to the equal effective waveguide layer sizes, e.g., *t*_*eff*_ = (*t*_1_, *t*_2_), which corresponds to the approximate resonance intensities depicted in Fig. 2a. Additionally, for a surface fluctuations thickness of (*t*_1_, *t*_2_) = (40, 80) nm, there are 12 field nodes at (${\lambda }_{1}^{{\text{E}}}$, ${\lambda }_{3}^{{\text{E}}}$) and 10 field nodes at (${\lambda }_{2}^{{\text{E}}}$, ${\lambda }_{4}^{{\text{E}}}$), calculated using the formula of 2*Λ*/*p*_12_. Note that the choice of using 500 nm and 600 nm periods in this study is unique and not typical. The resonance mode under TM polarization in Fig. 2d is clearly observed to be the result of a hybridization between the SP resonance mode generated at the interface of the metallic silver substrate and waveguide layer, as well as the WG resonance mode generated between the waveguide layer/grating and the surrounding air. In Fig. 2d, when (*t*_1_, *t*_2_) = (40, 80) nm, the field magnitude at ${\lambda }_{1}^{{\text{M}}}$ is larger. Similarly, when (*t*_1_, *t*_2_) = (80, 40) nm, the field magnitude at ${\lambda }_{4}^{{\text{M}}}$ is larger. It is important to note that the field values in the figures are expressed as ratios compared to the field magnitude of the incident light in air (|*E*_0_|). The results illustrate significant enhancements of the resonance fields^31–33^, with a factor exceeding 14 and 8 for TE and TM polarization respectively, as depicted in Fig. 2c,d. In conclusion, this study establishes that the strengths and local field enhancements of the doublet resonance modes can be independently regulated by manipulating the fluctuation thickness of the constitutive gratings.

**Figure 3:**
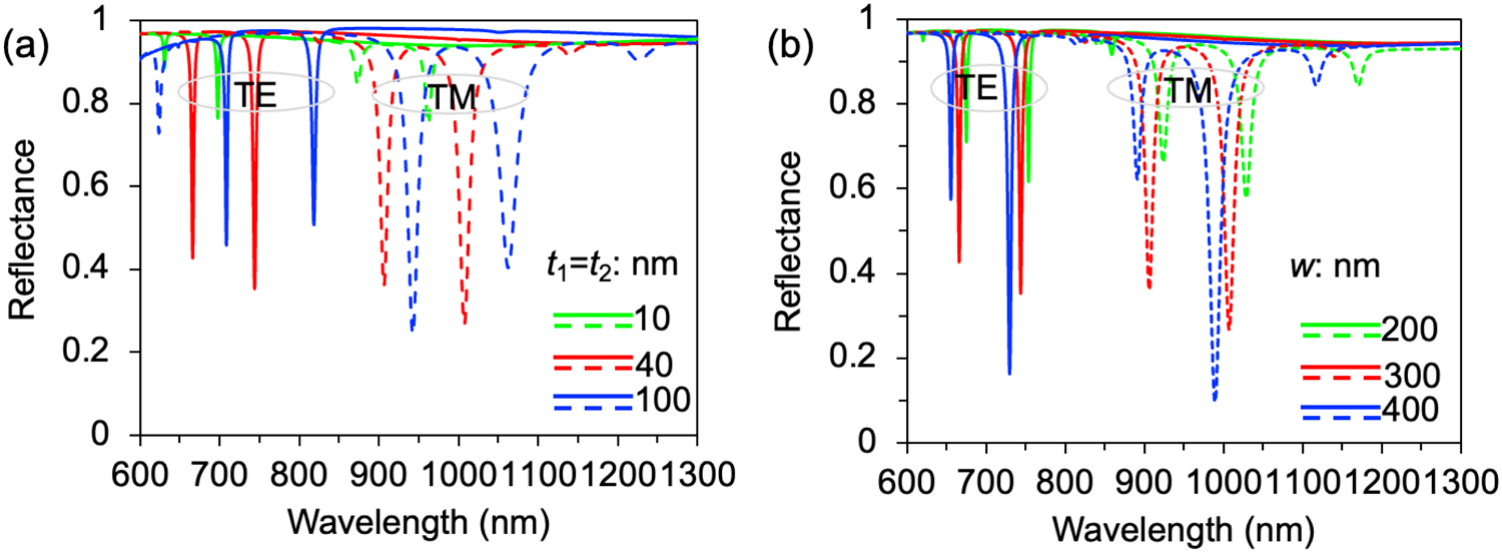
(**a**) Effects of surface fluctuation thickness (e.g., *t*_1_ = *t*_2_) on respective strengths of the doublet resonances for TE and TM polarizations. (**b**) Reflective Effects of relative width of the constitutive gratings under normal incidence of TE and TM polarized light, respectively. Here, (*p*_1_, *p*_2_) = (500, 600) nm and *t*_d_ = 100 nm.

To fully understand the observed doublet resonance mode in the structure, we further investigate a multiplexed dielectric rectangular grating with varying thicknesses and constitutive periods of (*p*_1_,* p*_2_) = (500, 600) nm, as shown in Fig. 3a. It is evident that twelve distinct resonant dips are observed for the TE and TM polarizations, respectively. The resonant dip shows a shallower and narrower profile with a metrology thickness of 10 nm, while for both TE and TM polarizations, the resonance wavelength position experiences a redshift with increasing gate thickness. In comparison to TM polarizations, TE polarization allows for achieving higher *QF* in resonance dips. Optimization of the dual-band resonance mode was achieved with a grating thickness of 40 nm; however, with an increase in the grating thickness to 100 nm, all resonant modes weaken and the intensity of the dual resonance mode reverses as shown in Fig. 3a. For grating thicknesses of 10 and 40 nm, the reflectance intensity in the shorter wavelength range is observed to be weaker than that in the longer wavelength range. One possible explanation could be the enhanced coupling of optical photons to the waveguide layer, leading to increased optical losses absorbed by the metal substrate layer. This phenomenon primarily arises from metals' tendency to absorb more light in the shorter wavelength range. We conducted an investigation on how different duty ratios of the constitutive dielectric rectangular gratings (*w* = 200, 300, and 400 nm) affect this phenomenon, as illustrated in Fig. 3b. Controlling the dimensional accuracy of mass production poses challenges, with errors typically ranging from a few nanometers to several nanometers. Our proposed structure demonstrates minimal movement in resonance position (Δ*λ* = 1 ~ 10 nm) and negligible variation in resonance intensity when subjected to rapid changes in grating size (Δ*w* = 100 nm). In Fig. 3b, compared to the case of *w* = 300 nm, the resonance dips weaken significantly (nearly halved) and exhibit red shifts for *w* = 200 nm. However, for w = 400 nm, the second dip becomes stronger while the first dip weakens. This could be attributed to smaller period gratings (*f* = *p*_1_-*w* = 100 nm) being covered by larger ones (*f* = *p*_2_-*w* = 200 nm) as the duty ratio width increases, resulting in additional enhancement of resonances with the assistance of smaller periods for the second dips. Another contributing factor to the redshift and improved reflectivity is the effective index of the gratings. For such a sub-wavelength grating, where the wavelength is larger than the grating period (*λ* >> *p*), only the zeroth-order wave propagates while the higher order modes are cutoff. Thus, using the nearly quasi-static (NQS) limit, effective index of the grating *n*_*geff*_ is the solution for two transcendental equations for TE and TM mode^30,34^:

1 $${n}_{geffTE}={n}_{geffTE0}^{2}+\frac{1}{3}{\left(\frac{\pi f\left(1-f\right)p}{\lambda }\right)}^{2}{\left({n}^{2}-1\right)}^{2}$$

2 $${n}_{geffTM}={n}_{geffTM0}^{2}+\frac{1}{4}{\left(\frac{\pi f\left(1-f\right)p}{\lambda }\right)}^{2}{n}_{geffTE0}^{2}{n}_{geffTM0}^{6}$$

3 $${n}_{geffTE0}={[\left(1-f\right)+f{n}^{2}]}^\frac{1}{2}$$

4 $${n}_{geffTM0}=\frac{n}{\sqrt{4-3f}}$$

where *f* is the fill factor defined as the ratio between the grating line widths (*p*-*w*) and period (*p*). The term " *n*_*geff*_ " refers to ${n}_{\left({t}_{1}, { t}_{2}\right)eff}$ in the equation. Based on Eq. (1), it can be known that the effective index of the gratings *n*_*geff*_ decreased with the increasing of *w,* which resulting in the red shift according to the grating equation [*n*_*geff*_ = *mλ*/*p*], where *m* is the diffraction order. For the TM polarization, the same situation observed according to Eq. (2). The above formula enables us to gain a deeper understanding and more accurate estimation of the impact that each parameter has on both the TE-polarized WG resonance mode and the TM-polarized hybrid SP resonance mode in conjunction with the WG-SP. This knowledge will facilitate better regulation of the resonant position and strength of the reflected dip. It can be concluded that this structure offers polarization-selective flexibilities in controlling the relative strength and local field enhancement of the multiplet resonance modes through adjustment of the dielectric rectangular grating components' relative duty ratio *w*.

**Figure 4:**
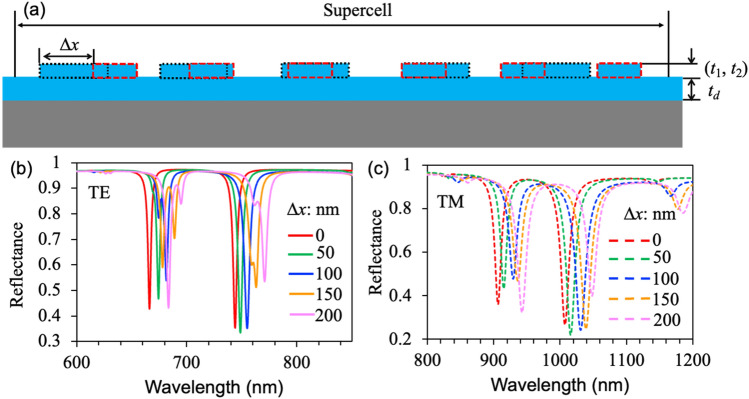
Effects of horizontal displacement (Δ*x*) on the characteristics of multiplexed gratings made from dielectric rectangular structures. (**a**) Surface profile of the gratings with constitutive periods of 500 and 600 nm for horizontal displacements. (**b**) and (**c**) Reflection spectra for TE and TM polarizations, respectively, are presented for gratings with constitutive periods of 500 and 600 nm at varying displacement (Δ*x*).

If a horizontal shift occurs in a grating from a doublet or triplet, it may result in a variation in the performance of the multiplexed grating. Therefore, we conducted an investigation on the effects of horizontal displacement (Δ*x*) on dielectric rectangular gratings with characteristics similar to those of constitutive doublet gratings. In Fig. 4a, the surface profile of the horizontal displacement doublet gratings is illustrated, with the red dotted line representing a period of *p*_1_ = 500 nm and the black dotted line representing a period of *p*_2_ = 600 nm. The reflection spectra in Fig. 4b demonstrate that as the horizontal displacement Δ*x* increases from 0 to 100 nm for the first resonance dips, they undergo splitting and weakening; however, there is minimal change observed for the second resonance dips. Upon reaching 200 nm, the first resonance dips become stronger compared to the second resonance dips. Furthermore, all resonances exhibit a red shift due to an increase in grating periods. For the TM polarization, as depicted in Fig. 4c, the resonance dips exhibit similar characteristics to those observed for TE polarization in Fig. 4b. However, a slightly broader FWHM and absence of dip splitting are observed with increasing horizontal displacement Δ*x*. This can be attributed to the coupling effect of SP. On the other hand, in TE polarization, the formation of a reflected dip is primarily caused by the refractive index modulation of the grating structure. Consequently, the reflection spectrum does not exhibit significant variability when controlling for resonant modes, except for the redshift and reflectance intensity swaps in TM polarization. Despite the considerable magnitude of the transverse displacement, its effect on the resonant mode remains negligible, thereby highlighting its crucial role in multi-band sensing, spectral detection, and other relevant applications.

**Figure 5:**
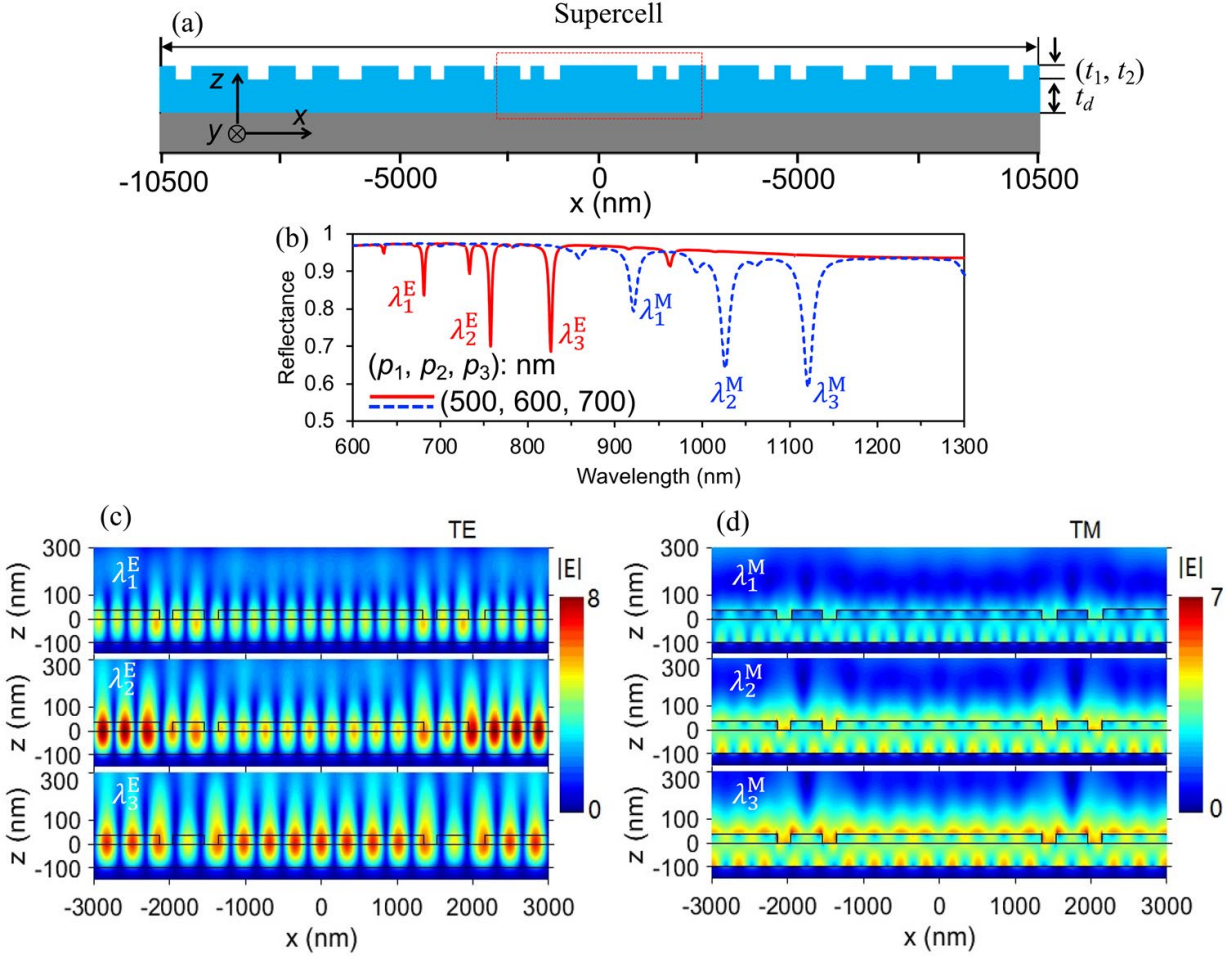
A simulation demonstration on resonance characteristics of a triplet dielectric rectangular grating. (**a**) Surface profile of a multiplexed dielectric grating with constitutive periods of 500, 600, and 700 nm. (**b**) Resonance characteristics in reflection spectra of the grating under normal incidence of TE- and TM- polarized light. (**c**) Corresponding field distributions (|*E*|) at the resonance wavelength.

Furthermore, we conducted an investigation on the multiplexed dielectric grating featuring triplet modes. The surface profile of such a grating with constitutive periods of (*p*_1_, *p*_2_, *p*_3_) = (500, 600, 700) nm reveals a significant increase in supercell size (Supercell pitch *Λ* = 21,000 nm), as depicted in Fig. 5a. The reflection spectra clearly exhibit resonance dips corresponding to the triplet reflectance at wavelengths of (${\lambda }_{1}^{E}$, ${\lambda }_{2}^{E}$, ${\lambda }_{3}^{E}$) = (681.2, 757.4, 826.2) nm and (${\lambda }_{1}^{M}$, ${\lambda }_{2}^{M}$, ${\lambda }_{3}^{M}$) = (921.1, 1026.1, 1120.6) nm for both TE- and TM-polarized light. The reflectance resonance dips exhibit additional spatial frequencies corresponding to virtual periods of the gratings, distinct from the real-space ones (*p*)^35^, as shown in Fig. 5b. The red rectangle depicted in the Fig. 5a represents a specific segment of the field distribution, facilitating its comprehensive analysis. In Fig. 5c,d, significant field enhancement is observed at the resonance positions on both the waveguide layer and dielectric grating surface^33^. Therefore, it can be concluded that despite the large pitch supercells limiting the intensity, triplet resonance modes can still be achieved.

### Application for multi-narrow-band sensing

**Figure 6:**
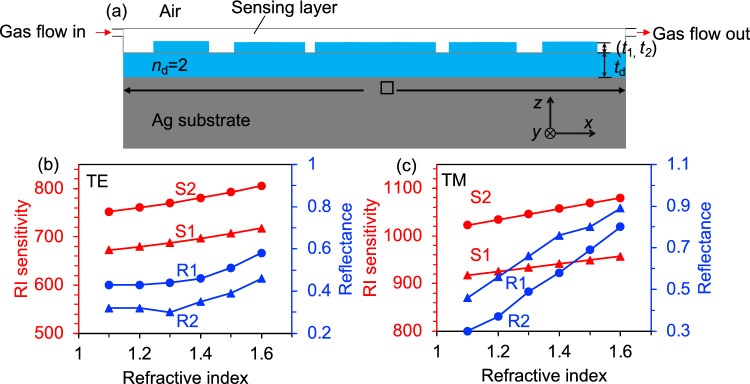
(**a**)Schematic of the grating gas sensor. Resonance wavelength shifts with the refractive index of surrounding environment and exhibits different sensing performances with (**b**) TE and (**c**) TM polarization under normal incidence. Here, (*p*_1_, *p*_2_) = (500, 600) nm and *t*_d_ = 100 nm, *t*_1_ = *t*_2_ = 40 nm.

In the above discussion, the proposed structure demonstrated the capability of achieving highly efficient narrow resonant dips in the near-infrared region for both TE and TM polarizations, making it suitable for multi-band refractive index sensors. The sensitivity of the refractive index sensor critically depends on the strength of the local field enhancement and the overlap of the fields. The advantage of our proposed structure is that it satisfies this criterion while bringing the sensing material into the structure, and therefore high sensitivity can be expected. To evaluate the performance of this multi-band refractive index sensor, we introduced gas with varying refractive indices (*n*) onto the surface of the grating. The resonance wavelengths of supercell gratings are influenced by their surrounding background medium, which holds significant potential for gas-sensing applications^36^. Based on this concept, we designed an integrated microfluidic channel gas sensor using multiplexed dielectric grating segments on a metal substrate, as illustrated in Fig. 6a. In this investigation, we focus on a dual-band structure induced by a grating of (*p*_1_, *p*_2_) = (500, 600) nm. The reflectance resonance dips and reflection efficiency for both TE and TM polarization are presented in Fig. 6b,c, illustrating a significant redshift in resonance wavelength with changes in ambient refractive index. This capability to achieve resolvable spectral tuning of reflectance enables the detection of even subtle variations in gas reflective index, making it suitable for application as a biological sensor to monitor changes in environmental conditions. Specifically, an increase in the gas refractive index from 1.1 to 1.6 leads to a redshift for TE polarization from 672.6 to 717.8 nm and from 751.8 to 805.7 nm, achieving a minimum FWHM of only 2 and 3 nm respectively, with a step interval Δ*λ* approximately equaling to 11 and 13 nm for S1 and S2 as illustrated in Fig. 6b. Similarly, for TM polarization, the resonance positions S1 and S2 shifted from 917 0.4 to 957 0.2 nm and from 1022 0.6 to 1079.6 nm, respectively, resulting in a minimum FWHM of only 8 and 9 nm, and a step interval Δ*λ* around 8 and 11 nm, respectively, as depicted in Fig. 6c.

This is the operational principle of our reflectance index sensor structure. A variation in the refractive index (RI) of the analyte, such as a gas, at the upper layer of a multiperiod composite grating induces a shift in formants, enabling detection and measurement of the analyte's RI by determining the formant position. RI sensitivity and Figure of Merit (FOM) are commonly employed to quantitatively assess the performance of reflectance index sensors. An ideal RI sensor should exhibit both high sensitivity and high FOM across a broad spectral range. The sensitivity of refractive index (nm/RIU) and FOM, defined by the formulas *S* = Δ*λ*/Δ*n* and FOM = *S*/FWHM, respectively. Where Δ*λ* represents the wavelength shift and Δ*n* denotes the refractive index change. The sensing performance, as depicted in Table 1, predominantly lies within the visible near infrared or infrared regions, making it challenging to achieve a broader spectral range simultaneously. Furthermore, most compound structures solely present sensing performance data for a single band and are subject to polarization limitations.

**Table 1:** RI sensing performances for multiplexed dielectric grating compound structures.

Reference	Spectral range (nm)	(*S*_1_*, S*_2_) nm/RIU	FOM
^37^	500–800	(470, 570)	(23, UR)
^38^	3000–14,000	TE/TM(1085, 1472)	UR
^39^	550–2000	TE/TM(853, UR)	(126, UR)
^40^	400–1000	TM(317, UR)	(8.3, UR)
In this work	All spectrum range	TE(110, 131)	TE(22, 26.2)
		TM(80, 114)	TM(5.7, 8.1)

In sensing applications, achieving high sensing performance remains a major challenge due to the instability of designed structures and complex fabrication processes. Although most of the reported compound sensing structures fulfill certain performance requirements (operating in either the near infrared or visible region, with a relatively wide FWHM), their sensing performance remains unreported (UR) and fail to meet polarization selection characteristics, significantly limiting their practical applicability^37,39–42^. For TM polarization with multiple independently regulated resonant modes, this structure has been used not only as a multi-band sensor or filter, but also in other devices to realize various aspects of micro and nanostructures. For instance, the dual-resonant mode structure is employed in photoluminescence devices where one resonance enhances the excitation signal while another resonance enhances the emitted signal^43^. This allows simultaneous enhancement and arbitrary independent regulation of both the excitation and emission signals, which is not possible with alternative structures. Thus, in addition to the aforementioned advantages, where a wide range of cost-effective optical components and sources are readily available, the proposed stable multi-band optical filter or refractive index sensor structure offers the advantage of a simplified geometry.

## Discussion

In summary, our investigation focuses on the utilization of multiplexed dielectric rectangular gratings to overcome the limitations of conventional optical resonators in controlling and enhancing light-matter interactions. We have successfully realized controllable and polarization-selective multiplet resonant modes. Unlike metallic gratings on metallic substrates, our proposed dielectric rectangular multiplex structure exhibits narrow-band multiplet resonant modes induced by WG and SP hybrid resonant modes, while maintaining polarization-selective in TE and TM polarizations. The electric field intensity (|*E*|) is demonstrated to enhance the understanding of resonant mechanisms and field overlapping properties, providing valuable insights. Additionally, multiplexed gratings with doublet or triplet SP hybrid resonant modes for TM polarization have been successfully showcased as independently tunable by adjusting the constitutive physical parameters. The refractive index sensing capabilities of the two resonance modes are subsequently investigated. In TM polarization, the hybrid SP resonance modes exhibit a calculated sensitivity of (80, 114) nm/RIU, resulting in FOM of (5.7, 8.1)/RIU. On the other hand, the WG resonance modes demonstrate even superior sensing performance in TE polarization with a sensitivity of (110, 131) nm/RIU and FOM values reaching (22, 26.2). These findings unveil that while the multiplexed dielectric rectangular grating structure possesses lower refractive index sensitivity compared to other structures, it offers a wider wavelength range and higher overall sensitivity. This structural configuration exhibits exceptional performance in refractive index sensing applications. The proposed multiplexed dielectric rectangular grating structure can be easily fabricated using lithography processes and offers a novel approach for designing multiple light-matter interactions that hold great potential in multi-band sensing, photodetectors, filters, and multispectral devices.

## Methods

The reflection characteristics and electric field distribution intensity are simulated using the finite difference time-domain method (FDTD)-method-based tool “commercial software FDTD Solutions, version: 1.8.3494” that is available from Lumerical, Canada^44^. In order to achieve comparable results in the simulation, we employed a technique for simplifying the three-dimensional (3-D) model into a two-dimensional representation. Moreover, utilizing 2-D simulation models offers significant time and computational resource savings compared to their 3-D counterparts, while also facilitating easier manufacturing processes^45,46^. In the numerical simulations, a mesh size of 2 nm is utilized. The light source consists of a plane wave with TM-polarized incident light propagating in the *x*-direction and TE-polarized incident light propagating in the *y*-direction. These waves are normally incident on the structure from the top side while being in air. The dielectric medium has a refractive index of *n* = 2. The simulation environment is bounded along the y-axis by perfectly matched layer (PML) boundary conditions, while periodic boundary conditions are considered in the *x*-direction. The substrate thickness is sufficient to effectively impede the propagation of incident phonons throughout the entire structure, given that the Ag substrate exceeds the skin depth with a permittivity characterized by the Drude model^47^. The permittivity of a metal (Ag) is defined by the following formula, which corresponds to the Drude model^33^:

5 $$\varepsilon ={\varepsilon }_{\infty }-\frac{{\omega }_{p}^{2}}{{\omega }^{2}+i\gamma \omega }$$

where ${\omega }_{\infty }$=3.7, ${\omega }_{p}$=9.1 eV = 1.37 × 10^16^ rad/s, $\gamma$= 0.018 eV = 0.27 × 10^14^ rad/s.

## Data Availability

The datasets generated and analyzed during the current study are available from the corresponding author on reasonable request.
